# Effects of enamel matrix derivative and transforming growth factor-β1 on human osteoblastic cells

**DOI:** 10.1186/1746-160X-7-13

**Published:** 2011-07-18

**Authors:** Daniela B Palioto, Thaisângela L Rodrigues, Julie T Marchesan, Márcio M Beloti, Paulo T de Oliveira, Adalberto L Rosa

**Affiliations:** 1 Department of Oral Maxillofacial Surgery and Periodontology, School of Dentistry of Ribeirão Preto - University of São Paulo, Av. do Café s/n, 14040-904 Ribeirão Preto, SP, Brazil; 2 Department of Morphology, Stomatology and Physiology, School of Dentistry of Ribeirão Preto - University of São Paulo, Av. do Café s/n, 14040-904 Ribeirão Preto, SP, Brazil

## Abstract

**Background:**

Extracellular matrix proteins are key factors that influence the regenerative capacity of tissues. The objective of the present study was to evaluate the effects of enamel matrix derivative (EMD), TGF-β1, and the combination of both factors (EMD+TGF-β1) on human osteoblastic cell cultures.

**Methods:**

Cells were obtained from alveolar bone of three adult patients using enzymatic digestion. Effects of EMD, TGF-β1, or a combination of both were analyzed on cell proliferation, bone sialoprotein (BSP), osteopontin (OPN) and alkaline phosphatase (ALP) immunodetection, total protein synthesis, ALP activity and bone-like nodule formation.

**Results:**

All treatments significantly increased cell proliferation compared to the control group at 24 h and 4 days. At day 7, EMD group showed higher cell proliferation compared to TGF-β1, EMD + TGF-β1 and the control group. OPN was detected in the majority of the cells for all groups, whereas fluorescence intensities for ALP labeling were greater in the control than in treated groups; BSP was not detected in all groups. All treatments decreased ALP levels at 7 and 14 days and bone-like nodule formation at 21 days compared to the control group.

**Conclusions:**

The exposure of human osteoblastic cells to EMD, TGF-β1 and the combination of factors *in vitro *supports the development of a less differentiated phenotype, with enhanced proliferative activity and total cell number, and reduced ALP activity levels and matrix mineralization.

## Introduction

Periodontal regeneration is a complex series of cell and tissue events that include cell adhesion, migration, and extracellular matrix (ECM) protein synthesis and secretion. Phenotypic expression depends on cell interactions with ECM proteins, which regulate cell signaling events and ultimately gene expression[1]. The ECM proteins are, therefore, key factors that influence the regenerative capacity[2]. However, to date, it remains undefined which factors would determine the maximum regenerative capacity.

Enamel matrix derivative (EMD) has been used in various clinical applications aiming to promote periodontal tissue regeneration. The rationale for such application is based on the expression of enamel matrix proteins during the initial phases of root formation, which has been associated with cementoblast differentiation[3,4]. In addition, the use of EMD in various experimental and clinical protocols has been demonstrated to positively affect not only new cementum formation but also bone regeneration[5-8]. However, some controversial results in terms of new bone formation has also been described in the literature[9].

Despite clinical evidences supporting a positive effect of EMD on periodontal regeneration and *in vitro *observations on how EMD affects PDL fibroblasts[10] and osteoblast functions[11], it is still to be clarified the mechanisms by which EMD stimulates different periodontal cell types and differentiation stages. It seems to be well determined that EMD upregulates proliferation of PDL fibroblasts [10,12,13], cementoblasts[14], follicle cells[15], and osteoblasts[16]. The controversial results are, indeed, focused on how, and if so, EMD promotes cell differentiation in various cell types. For instance, while the addition of EMD in MG63 cell cultures results in the upregulation of osteocalcin and TGF-β1[17], it does not affect cell differentiation in other osteoblastic cell lines[18].

Althought Gestrelius et al. [12] demonstrated that EMD has no growth factors in its composition, others have shown that EMD may act as a natural and efficient drug delivery system for growth factors including TGF-β1[19]. Additionaly, EMD can stimulate the production of TGF-β1 by cells[17]. Indeed, PDL cells express high levels of endogenous TGF-β1 on the presence of EMD[20-22], raising the hypothesis that the action of EMD would be mediated by growth factors found in its composition or in the culture medium modified by cells under EMD exposure[15].

The interactions between growth factors and precursor cells are key factors in the process of periodontal healing and regeneration[23] and the association of growth factors seems to synergistically affect the regenerative process[24-27]. Because the effects of the association of EMD with growth factors and other proteins are still little explored, and considering that TGF-β1 regulates various cellular activities and has been demonstrated to affect osteoblastic cell behavior, the present study aimed to evaluate the effects of EMD, exogenous TGF-β1 and the association of such factors on key parameters of the development of the osteogenic phenotype in human alveolar bone-derived cell cultures.

## Materials and methods

### Cell culture

Human alveolar bone fragments (explants) were obtained from adult healthy donors (ranging from 15 to 25 years old), using palatal/lingual and/or interradicular alveolar bone associated with either premolars or third molars extracted for orthodontic reasons, with clinically healthy periodontium. Osteoblastic cells were obtained from these explants by enzymatic digestion using collagenase type II (Gibco - Life Technologies, Grand Island, NY) as described by Mailhot and Borke[28]. Importantly, to avoid contamination with periosteal, periodontal ligament, and gingival cells, bone fragments were scrapped and the first 2 digestions were discarded. Primary cells were cultured in α-minimum essential medium (α-MEM - Gibco), supplemented with 10% fetal bovine serum (FBS - Gibco), 50 μg/mL gentamicin (Gibco), 0.3 μg/mL fungizone (Gibco), 10^-7 ^M dexamethasone (Sigma, St. Louis, MO), 5 μg/mL ascorbic acid (Gibco), and 7 mM β-glycerophosphate (Sigma). Such osteogenic culture condition supports the development of the osteoblastic phenotype[29,30].

Subconfluent cells in primary culture were harvested after treatment with 1 mM ethylenediamine tetraacetic acid (EDTA - Gibco) and 0.25% trypsin (Gibco) and subcultured cells under osteogenic culture condition were used in all experiments. The progression of the subcultured cells and the acquisition of the osteoblastic phenotype have been well characterized by the work of de Oliveira et al. [31]. During the culture period, cells were incubated at 37°C in a humidified atmosphere of 5% CO_2 _and 95% air; the medium was changed every three or four days. All experiments were performed using three different sets of subcultures, and each experiment conducted in quadruplicate. All patients were informed about the study's purpose before they consented to participate. The local Research Ethics Committee approved the protocol.

### Treatments

Emdogain gel (EMD - Biora, Malmo, Sweden) was dissolved in acidic water, pH 5.9, whereas TGF-β1 (Sigma Chemical Co., St. Louis, MO, USA) was dissolved in acetonitrile plus trifluoracetic acid (Sigma). Both solutions were aliquoted and stored at -70°C. Two concentrations had to be chosen because the osteoblastic cell subculture would not allow a more extensive experimental design than the one proposed herein. Thus, based on previous studies[10,32], treatment with EMD and TGF-β1 was performed at concentrations of 100 μg/mL and 5 ng/mL, respectively. Four experimental conditions were established: 1) medium supplemented with 10% FBS (control); 2) 100 μg/mL EMD in medium supplemented with 10% FBS (EMD group); 3) 5 ng/mL TGF-β1 in medium supplemented with 10% FBS (TGF-β1); 4) combination of 100 μg/mL EMD and 5 ng/mL TGF-β1 in medium supplemented with 10% FBS (EMD+TGF-β1 group). The final pH for all groups was in the 7.2-7.4 range. A negative control was not possible because culture medium with either no FBS or a minimum concentration of FBS did not support the progression of the osteoblastic cell cultures (data not shown).

### Cell growth assay

The cell growth assay was performed using a modified method of Coletta et al. (1998)[33]. Osteoblastic cells were plated in a 24-well culture plate (Corning Inc., NY, USA) at a density of 20,000 cells/well in 1 mL of α-MEM supplemented with 10% FBS (Gibco), 50 μg/mL gentamicin (Gibco), 0.3 μg/mL fungizone (Gibco), 10^-7 ^M dexamethasone (Sigma), 5 μg/mL ascorbic acid (Gibco), and 7 mM β-glycerophosphate (Sigma). The cells were allowed to attach and spread for 24 h, and then washed with PBS and cultured in serum-free α-MEM for an additional 24 h. After treatments with the four experimental conditions for four and seven days, cells were enzymatically (1 mM EDTA, 1.3 mg/mL collagenase type II, and 0.25% trypsin - Gibco) detached. Aliquots of these solutions were incubated for 5 min with the same volume of trypan blue and directly counted in a hemocytometer (Fisher Scientific, Pittsburgh, PA, USA). For each time point, total cell number (×10^4^/well) was determined, which included trypan blue-stained cells.

### Bromodeoxyuridine-labeling (BrdU) index

Effect of EMD, TGF-β1 and the combination of both on osteoblastic cells proliferation was assessed by direct counting of cell number and BrdU incorporation into DNA. The BrdU is detecting in the tissue through primary antibodies. These primary antibodies are then labeled with a secondary antibody tagged with a substrate for diaminobenzidine (DAB, Nunc International, Naperville, IL, USA)[34]. The substitution of an endogenous DNA base, thymidine, with the BrdU analogue ensures specific labeling of only the dividing cells during S-phase (DNA synthesis). Osteoblastic cells were plated on 8-well glass culture chamber slides (Nunc International, Naperville, IL, USA) at a density of 20,000 cells/well in 500 μl of α-MEM supplemented with 10% FBS (Gibco), 50 μg/mL gentamicin (Gibco), 0.3 μg/mL fungizone (Gibco), 10^-7 ^M dexamethasone (Sigma), 5 μg/mL ascorbic acid (Gibco), and 7 mM β-glycerophosphate (Sigma), and were incubated at 37°C and 5% CO_2_. Following 24 h of serum starvation, cells were exposed to the four experimental culture conditions for 24 h. After treatment, cells were incubated with BrdU (diluted 1:1,000) for 1 h under the same conditions, washed in PBS and fixed in 70% ethanol for 15 min. BrdU incorporation in proliferating cells was revealed using immunohistochemistry (Amershan Pharmacia Biotech Inc., Piscataway, NJ). Briefly, the anti-5-bromo-2'-deoxyuridine monoclonal antibody, diluted 1:100 in nuclease with deionized water, were added to the wells and incubated for 1 h. The wells were then washed three times with 500 μL of PBS and the peroxidase anti-mouse IgG2a (15:1,000) were added to the wells and incubated for 1 h. After another washing step, the reaction was developed with 0.6 mg/mL of 3,3'-diaminobenzidine tetrahydrochloride (Sigma) containing 1% H_2_O_2 _and 1% DMSO for 5 min at 37°C. The cells were then stained with Crazzi hematoxylin and examined under transmitted light microscopy. The BrdU labeling index, expressed as the percentage of cells labeled with BrdU, was determined by counting 1,500 cells using an image analysis system (Kontron 400, Zeiss, Eching bei Munich, Germany).

### Fluorescence labeling

For immunofluorescence labeling of noncollagenous matrix proteins, cells were treated with the four experimental culture conditions for five days. At day 5, cells were fixed for 10 min at room temperature (RT) using 4% paraformaldehyde in 0.1 M phosphate buffer (PB), pH 7.2. After washing in PB, they were processed for immunofluorescence labeling[31]. In addition, cell adhesion and spreading were morphologically evaluated by direct fluorescence with fluorophore-conjugated probes. Briefly, cells were permeabilized with 0.5% Triton X-100 in PB for 10 min followed by blocking with 5% skimmed milk in PB for 30 min. Primary monoclonal antibodies to bone sialoprotein (anti-BSP, 1:200, WVID1-9C5, Developmental Studies Hybridoma Bank, Iowa City, IA, USA), alkaline phosphatase (anti-ALP, 1:100, B4-78, Developmental Studies Hybridoma Bank), and osteopontin (anti-OPN, 1:800, MPIIIB10-1, Developmental Studies Hybridoma Bank) were used, followed by a mixture of Alexa Fluor 594 (red fluorescence)-conjugated goat anti-mouse secondary antibody (1:200, Molecular Probes) and Alexa Fluor 488 (green fluorescence)-conjugated phalloidin (1:200, Molecular Probes), which labels actin cytoskeleton. Replacement of the primary monoclonal antibody with PB was used as control. All antibody incubations were performed in a humidified environment for 60 min at RT. Between each incubation step, the samples were washed three times (5 min each) in PB. Before mounting for microscope observation, samples were briefly washed with dH_2_O and cell nuclei stained with 300 nM 4', 6-diamidino-2-phenylindole, dihydrochloride (DAPI, Molecular Probes) for 5 min. After mounting with an antifade kit (Prolong, Molecular Probes), the samples were examined under epifluorescence using a Leica DMLB light microscope (Leica, Bensheim, Germany), with N Plan (X2.5/0.07, X10/0.25, X20/0.40) and HCX PL Fluotar (X40/0.75, X100/1.3) objectives, outfitted with a Leica DC 300F digital camera. The acquired digital images were processed with Adobe Photoshop software (version 7.0.1, Adobe Systems).

### Total protein synthesis

Osteoblastic cells were plated in 24-well culture plates at a density of 20,000 cells/well in 2 mL of α-MEM supplemented with 10% FBS (Gibco), 50 μg/mL gentamicin (Gibco), 0.3 μg/mL fungizone (Gibco), 10^-7 ^M dexamethasone (Sigma), 5 μg/mL ascorbic acid (Gibco), and 7 mM β-glycerophosphate (Sigma) at 37°C in a humidified atmosphere with 5% CO_2_. Following serum starvation, cells were exposed to the four experimental culture conditions described previously for seven and fourteen days. Media was changed and supplemented every three or four days. Total protein content was determined using a modification of the Lowry method. Briefly, proteins were extracted from each well with 0.1% sodium lauryl sulphate (Sigma) for 30 min, resulting in a lysates of the cells, and mixed 1:1 with Lowry solution (Sigma) for 20 min at RT. The resulting solution was diluted in Folin and Ciocalteau's phenol reagent (Sigma) for 30 min at RT. Absorbance was measured at 680 nm using a spectrophotometer (Cecil CE3021, Cambridge, UK). The total protein content was calculated from a standard curve and expressed as μg/mL.

### Alkaline phosphatase activity

Osteoblastic cells were plated in 24-well culture plates at a density of 20,000 cells/well in 2 mL of α-MEM supplemented with 10% FBS (Gibco), 50 μg/mL gentamicin (Gibco), 0.3 μg/mL fungizone (Gibco), 10-7 M dexamethasone (Sigma), 5 μg/mL ascorbic acid (Gibco), and 7 mM β-glycerophosphate (Sigma) at 37°C in a humidified atmosphere with 5% CO_2_. Following serum starvation, cells were exposed to the four experimental culture conditions described previously for seven and fourteen days. Media was changed and supplemented every three or four days. Alkaline phosphatase (ALP) was extracted from each well with 0.1% sodium lauryl sulphate (Sigma) for 30 min, resulting in a lysates of the cells ALP activity was measured as the release of thymolphthalein from thymolphthalein monophosphate using a commercial kit (Labtest Diagnostica, MG, Brazil). Briefly, 50 μl thymolphthalein monophosphate was mixed with 0.5 ml 0.3 M diethanolamine buffer, pH 10.1, and left for 2 min at 37°C. The solution was then added to 50 μl of the lysates obtained from each well for 10 min at 37°C. For color development, 2 ml 0.09 M Na_2_CO_3 _and 0.25 M NaOH were added. After 30 min, absorbance was measured at 590 nm and ALP activity was calculated from a standard curve using thymolphthalein to give a range from 0.012 to 0.4 μmol thymolphthalein/h/ml. Data were expressed as ALP activity normalized for total protein content at 7 and 14 days.

### Mineralized bone-like nodule formation

Osteoblastic cells were plated in 24-well culture plates at a density of 20,000 cells/well in 2 mL of α-MEM supplemented with 10% FBS (Gibco), 50 μg/mL gentamicin (Gibco), 0.3 μg/mL fungizone (Gibco), 10^-7 ^M dexamethasone (Sigma), 5 μg/mL ascorbic acid (Gibco), and 7 mM β-glycerophosphate (Sigma) at 37°C in a humidified atmosphere with 5% CO_2_. Following serum starvation, cells were exposed to the four experimental culture conditions described previously with differentiation medium for 21 days. Media was changed and supplemented every three or four days. At day 21, cultures were washed in PBS and fixed with 10% formaldehyde in PBS, pH 7.2, for 16 h at 4°C. The samples were then dehydrated in a graded series of ethanol and stained with 2% Alizarin red S (Sigma), pH 4.2, for 8 min at RT. Using an inverted light microscope (X10 objective; Carl Zeiss, Jena, Germany), equipped with a digital camera (Canon EOS Digital Rebel Camera, 6.3 Megapixel CMOS sensor, Canon USA Inc., Lake Success, NY, USA), the formation of mineralized areas was analyzed. Ten microscopic fields in each sample were randomly selected and the mineralized area was measured as a percentage area of the well using an image analyzer (Image Tool; University of Texas Health Science Center, San Antonio, TX, USA).

### Statistical analysis

Data represent the pooled results of three independent experiments. Each experiment was conducted using cells of a single donor. All experiments were performed in quadruplicate for each set of subculture. All results are presented as mean ± standard deviation, and the non-parametric Kruskal-Wallis test for independent samples was used for statistical analyses. If the result of the Kruskal-Wallis test was significant (*P <*0.05), the Fischer's test for multiple comparisons, computed on ranks rather than data, was performed[35].

## Results

### Effect of EMD, TGF-β1 or both on cell proliferation and total cell number

**Figure 1:**
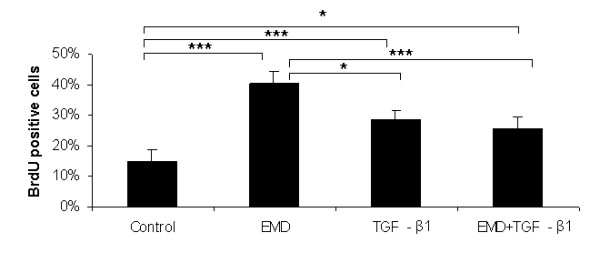
**Effect of EMD, TGF-β1 and the combination of both factors on cell proliferation by means of BrdU-labeling at 24 h post-treatment**. *P < 0.05; **P < 0.01; ***P < 0.001.

**Figure 2:**
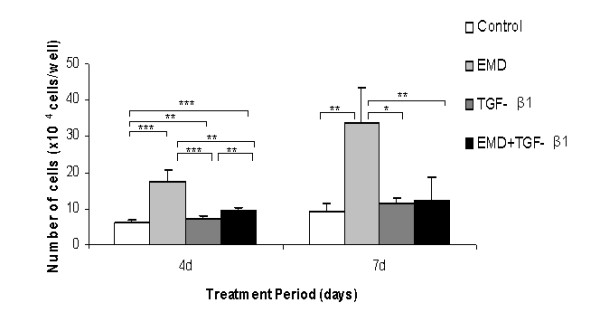
**Effect of EMD, TGF-β1 and the combination of both factors on cell growth**. All treatments showed an increase in cell proliferation. The EMD proliferation rate was higher than the positive control at days 4 and 7. *P < 0.05; **P < 0.01; ***P < 0.001.

Nuclear immunoreactivity for BrdU was clearly noticed in osteoblastic cells under all treatments. Both treatments and their combination affected the proliferation at the first 24 hours of experiments compared to the control (EMD, *P *< 0.001; TGF-β1, *P *< 0.001; EMD + TGF-β1, *P *< 0.05) (Figure 1). In addition, treatment with EMD significantly increased total cell number compared to TGF-β1 (*P *< 0.05) and the combination of the factors (*P *< 0.001). Treatments with EMD, TGF-β1 and EMD+TGF-β1 significantly increased total cell number at day 4 compared to the control (*P *< 0.001, *P *< 0.01, and *P *< 0.001, respectively); the treatment with only EMD resulted in higher values compared to the TGF-β1 treatment (*P *< 0.001) and the combination of the factors (*P *< 0.01), whereas total cell number for EMD+TGF-β1 was significantly higher compared to TGF-β1 (*P *< 0.01). On day 7, no statistical differences among TGF-β1, EMD+TGF-β1 and control groups were detected. However, all these groups showed a significantly lower number of cells compared to the EMD group (control, *P *< 0.01; TGF-β1, *P *< 0.05; EMD + TGF-β1, *P *< 0.01) (Figure 2).

### Cellular morphology and indirect immunofluorescence for localization of noncollagenous matrix proteins

**Figure 3:**
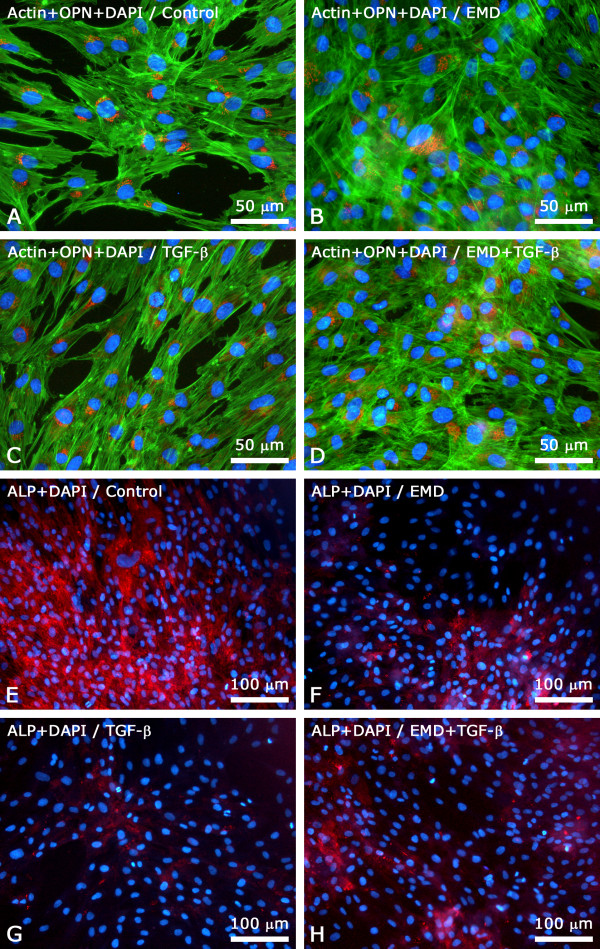
**Epifluorescence at day 5 post-treatment with the factors**. (A-D) Immunolabeling for osteopontin (OPN, red fluorescence) was mainly cytoplasmic, in perinuclear area and in punctate deposits. Cell-associated green fluorescence reveals actin cytoskeleton (Alexa Fluor 488-conjugated phalloidin), whereas blue fluorescence indicates cell nuclei (DAPI - DNA staining). No major differences were noticed among groups in terms of labeling pattern and fluorescence intensity for OPN. (E-H) Immunolabeling for alkaline phosphatase (ALP, red fluorescence) was more intense for the positive control compared to the treated groups.

Epifluorescence of actin cytoskeleton labeling revealed that cells were adherent and spread, showing a polygonal elongated morphology, with focal areas of multilayer formations (Figure 3A-D). Indirect immunofluorescence using a primary antibody anti-OPN showed that such protein was expressed in the majority of cells, mostly in the perinuclear area suggestive of Golgi apparatus, and in a dot pattern throughout the cytoplasm. No differences in terms of OPN labeling pattern and fluorescence intensities among control and EMD, TGF-β1 e EMD+TGF-β1 groups were noticed; for all groups, no extracellular OPN labeling was detected (Figure 3A-D). Immunolabeling for ALP was more intense for control than for the treated groups, with a labeling pattern characterized by punctate deposits throughout the cell surface and cytoplasm (Figure 3E-H). At day 5, no bone sialoprotein labeling was detected for all groups (data not shown).

### Effects of EMD, TGF-β1 or both on total protein synthesis, ALP activity, and mineralized matrix formation

**Figure 4:**
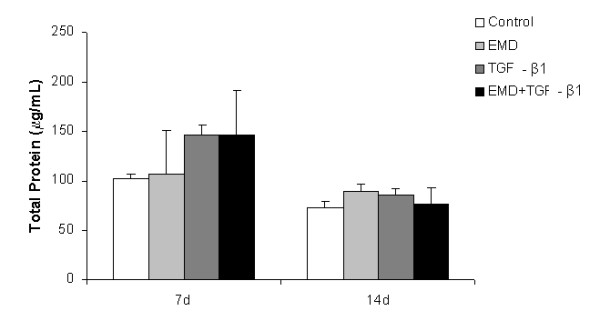
**Total protein content at 7 and 14 days**. The values (μg/mL) are expressed as mean ± SD of representative results of three separate experiments in cell cultures established from three different patients, performed in quadruplicate for each treatment. There were no statistically significant differences among groups (*P *> 0.05).

**Figure 5:**
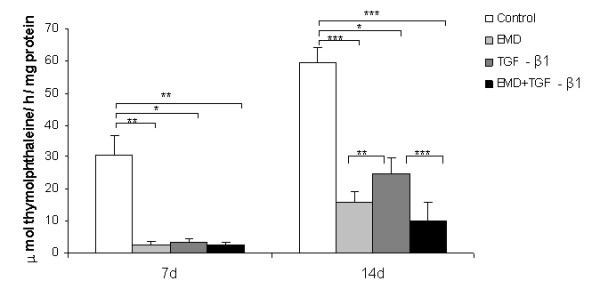
**ALP activity at 7 and 14 days**. The results are expressed as μmol thymolphthalein/h/mg protein. The values are expressed as mean ± SD of representative results of three separate experiments in cell cultures established from three different patients, performed in quadruplicate for each treatment. *P < 0.05; **P < 0.01; ***P < 0.001.

**Figure 6:**
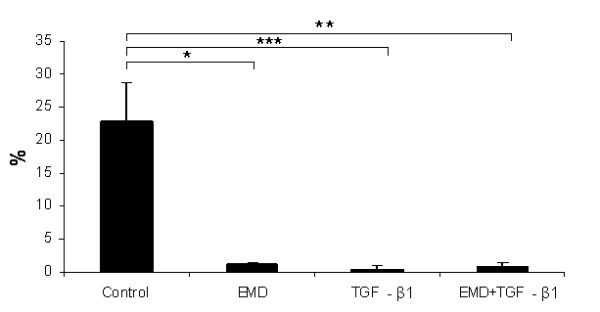
**Alizarin red S stained areas of osteoblastic cell cultures treated with 100 μg/mL EMD, 5 ng/mL TGF-β1, and 100 μg/mL EMD plus 5 ng/mL TGF-β1, at 21 days**. Percentage of stained areas was significantly higher for non-treated cultures. *P < 0.05; **P < 0.01; ***P < 0.001.

**Figure 7:**
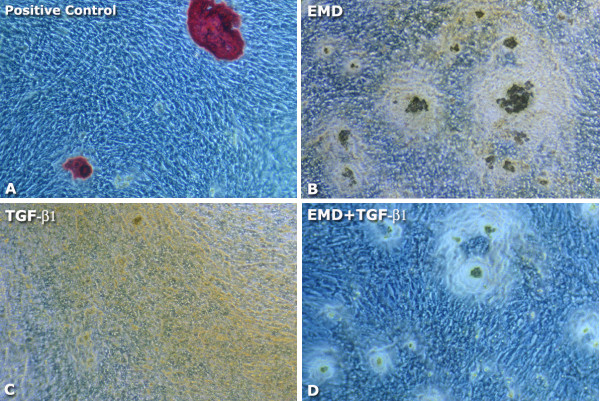
**Light microscopy of Alizarin red S stained-osteoblastic cell cultures: (A) control group; (B) 100 μg/mL EMD; (C) 5 ng/mL TGF-β1; (D) 100 μg/mL EMD plus 5 ng/mL TGF-β1**. Phase contrast, ×10 objective.

Total protein synthesis was not significantly affected by the treatments (*P *> 0.05) (Figure 4); however, a tendency for greater values of total protein was clearly seen at day 7 for all treated groups and for the EMD group at day 14. ALP activity was negatively affected by EMD, TGF-β1 and EMD+TGF-β1 treatments compared to the control both at days 7 and 14. On day 14, the treatments with EMD and EMD+TGF-β1 exhibited lower ALP activity than TGF-β1 group (*P *< 0.01 and *P *< 0.001, respectively) (Figure 5). At day 21, matrix mineralization was significantly higher for the control group compared to EMD (*P *< 0.05), TGF-β1 (*P *< 0.001) and EMD+TGF-β1 groups (*P *< 0.01) (Figures 6 and 7).

## Discussion

The exposure of human osteoblastic cells to EMD, TGF-β1 and EMD+TGF-β1 resulted in early increased cell proliferation, and reduced ALP activity and matrix mineralization. The present results are corroborated by several works that observed EMD stimulation of the proliferative capacity of both osteoblastic cells[14,16,36] and PDL fibroblasts[10,12,13,20,22]. In contrast to PDL fibroblast response to EMD, which shows signs of matrix mineralization when EMD are used even at earlier time points[13], osteoblastic cell cultures seem to be inhibited in terms of osteogenic differentiation. Interestingly, the association of EMD and exogenous TGF-β1 did not alter the osteogenic potential of the cultures.

Although the results of the present study point toward the development of a less differentiated osteoblastic phenotype when cells were exposed to EMD, TGF-β1 or EMD+TGF-β1, no morphologic differences were observed among the groups. Cell morphology was considered within the typical features of human alveolar bone-derived cells cultured on plain conventional substrates, showing an elongated polygonal shape[31,37,38]. None of the treatments supported the development and progression of stelate-like shaped cells, with thin and elongated cytoplasmic extensions, which could be indicative of less differentiated phenotypes[31].

The total protein content showed a tendency to be increased during the initial periods of cultures for all the treatments comparing to control, which could be due to the increased number of cells at the end of the proliferative phase. It has been demonstrated in various cell types that EMD seems to augment total protein production and collagen content[12,18].

It has been well-established that there is an inverse relationship between cell proliferation and cell differentiation for the osteoblast lineage; as the proliferative capacity increases, the cell differentiation decreases. Indeed, full expression of the osteoblast phenotype leads to terminal cell cycle exit[39,40]. In the present study, two multifunctional noncollagenous matrix proteins (OPN and BSP) with a role in the matrix mineralization process were used as osteoblastic cell differentiation markers[41-44]. OPN had a similar distribution and fluorescence intensities in cultures of all groups at day 5 post-treatments, which is in agreement with the biphasic pattern of expression (at days 5 and 14)[42] and supports the interpretation of the presence of less differentiated osteoblastic cells[45]. Since BSP is a marker of initial osteoblast differentiation, the absence of BSP labeling at day 5 post-treatment and in control cultures could indicate that none of the treatments were able to promote the early expression of this matrix protein. Based on published data, the effect of EMD in osteoblastic cells seems to be dependent on cell type and culture condition and to act in a dose-dependent manner[46,47]. Hama et al. [48], working with fetal rat calvarial cells, has reached similar results as the ones found in the present study. EMD decreased, in a dose dependent manner, osteocalcin and core binding proteins expression, ALP activity, and bone-like nodule formation. They also sought to determine the possible role of TGF-β1 on these effects by inhibiting its expression. Treatment with TGF-β1 antibody partly restored the inhibitory effect of EMD on ALP activity. Conversely, in our work human osteoblastic cells were sensitized with exogenous TGF-β1 and the same inhibitory effect on osteoblastic differentiation was noticed.

Although the roles of ALP during the process of matrix mineralization are still not fully clarified, it has been proposed that such enzyme generates the phosphate needed for hydroxyapatite formation. In addition, ALP has also been hypothesized to hydrolyze pyrophosphate, a mineralization inhibitor, in order to facilitate mineral precipitation and growth[31]. In the present study, a significant decrease in ALP activity at days 7 and 14 post-treatment with EMD, TGF-β1 or EMD+TGF-β1 was associated with reduced ALP immunodetection, a finding that is consistent with increased cell proliferation and reduced osteogenic potential of the cultures[31]. Indeed, significantly reduced mineralization levels were detected for all treated groups compared to control. The treatments likely delayed or limited the matrix mineralization process due to the lower levels of ALP activity.

TGF-β1 has been recognized as a molecule that acts on the proliferative capacity of osteoblastic cells but not on osteoblast activities, which include osteoid matrix production and mineralization. McCauley & Somerman [49] demonstrated that TGF-β1 inhibits the formation of mineralized nodules *in vitro*. In addition, TGF-β1 expressed by platelets in fracture sites or by osteoclasts during bone remodeling may stimulate the formation of an osteoid matrix with no mineral phase, which could be possibly related to the lower levels of ALP activity[50].

Finally, considering that the use of EMD and TGF-β1 has been proposed as a strategy to support periodontal tissue regeneration, the present *in vitro *results show an inhibitory effect on cell differentiation and cell-mediated matrix mineralization when human osteoblastic cells are exposed to either EMD, TGF-β1 or the combination of both. Although it is difficult to extrapolate the *in vitro *findings to the *in vivo *situation, we may speculate from these results that new bone formation in the context of periodontal regeneration could not be as prominent as dental cementum and periodontal ligament regeneration.

## Conclusion

Within the limits of the present study, the exposure of human osteoblastic cells to EMD, TGF-β1 and the combination of factors *in vitro *supports the development of a less differentiated phenotype, with enhanced proliferative activity and total cell number, and reduced ALP activity levels and matrix mineralization.

## Competing interests

The authors declare that they have no competing interests.

## Authors' contributions

DBP designed the research. MMB and ALR established the cell culture protocol. TLSR, JTM and MMB performed the research. DBP and PTO analysed the data. DBP and PTO wrote the manuscript. All authors read and approved the final manuscript.
